# Supplementary material for: An Optimized Transformation Protocol for Escherichia coli BW3KD with Supreme DNA Assembly Efficiency
Source: Microbiol Spectr. 2022 Nov 1;10(6):e02497-22. doi: 10.1128/spectrum.02497-22 (PMC9769673; doi:10.1128/spectrum.02497-22)
Supplement: Supplemental file 1 — Supplemental material. Download spectrum.02497-22-s0001.pdf, PDF file, 0.6 MB [file spectrum.02497-22-s0001.pdf]

An optimized transformation protocol for *Escherichia coli* BW3KD with supreme DNA assembly efficiency

Yuqing Yang[1,2], Menghui Liu[1], Tianqi Wang[1], Qian Wang[1], Huaiwei Liu[1], Luying Xun[1,3], Yongzhen Xia[1*]

[1]State Key Laboratory of Microbial Technology, Shandong University, Qingdao, 266237, People's Republic of China

[2]Institute of Marine Science and Technology, Shandong University, Qingdao 266237, People's Republic of China

[3]School of Molecular Biosciences, Washington State University, Pullman, WA, 99164-7520, USA.

* Correspondence could be addressed to Y. Xia. Tel: +86-532-58631572; Email: xiayongzhen2002@sdu.edu.cn, Tel: +86-532-58631501

Supplementary Materials and Methods

The detailed preparation and transformation protocols of TSS-HI

1. Solutions

10% PEG3350 (Sigma-Aldrich, cat. no. P4338), 5% DMSO (Sigma-Aldrich, cat. no. D8418, purify≥99.9%), 10% Glycerol, 20 mM$MgCl_2$ (Sigma-Aldrich, cat. no. M8266) were mixed with 2× fresh LB medium. The pH was adjusted to 6.1 with 6 M HCl. 4 M $MnCl_2$ was added to a final concentration of 140 mM. Distilled water was added to diluted the modified LB medium to bring it to 1×. To avoid precipitation, the $MnCl_2$ solution should sterilize separately. The TSS-HI buffer was aliquoted into 1 mL and stored at -20°C before use.

The KCM buffer contained 0.5 M KCl (Sangon Biotech, cat. no. A100395), 150 mM $CaCl_2$ (Sigma-Aldrich, cat. no. 5670), 250 mM $MgCl_2$ (Sigma-Aldrich, cat. no. M8266), and sterilized at 121°C, 20 min. The solution was stored at 4°C.

2. The preparation of competent cells

A fresh single colony of BW3KD was inoculated into 4 mL of LB medium and incubated at 37°C overnight. Then, 1% of the culture was transferred to 50 mL of fresh LB medium and cultured at 37°C until OD_{600} of 0.5. The cells were harvested by centrifuging at 4,000 × *g* at 4 °C for 10 min, washed with 1 mL chilled TSS-HI buffer once, and resuspended in 1 mL of chilled TSS-HI buffer. Then the mixture was chilled on ice for 10 min, and aliquoted into 30 μL per tube for use or storage at -80 °C.

3. The transformation

Five μL of 5 x KCM was mixed with DNA and H_2O to total 25 μL mixture. The mixture was gently mixed with 25 μL competent cells and incubated on ice for 30 min. The cells were heat-shocked at 42°C water for 90s and placed on ice. 250 μL of fresh LB medium was added for cell recovery at 37°C for 1 hour. The recovered culture was spread onto the LB plates with the indicated antibiotics.

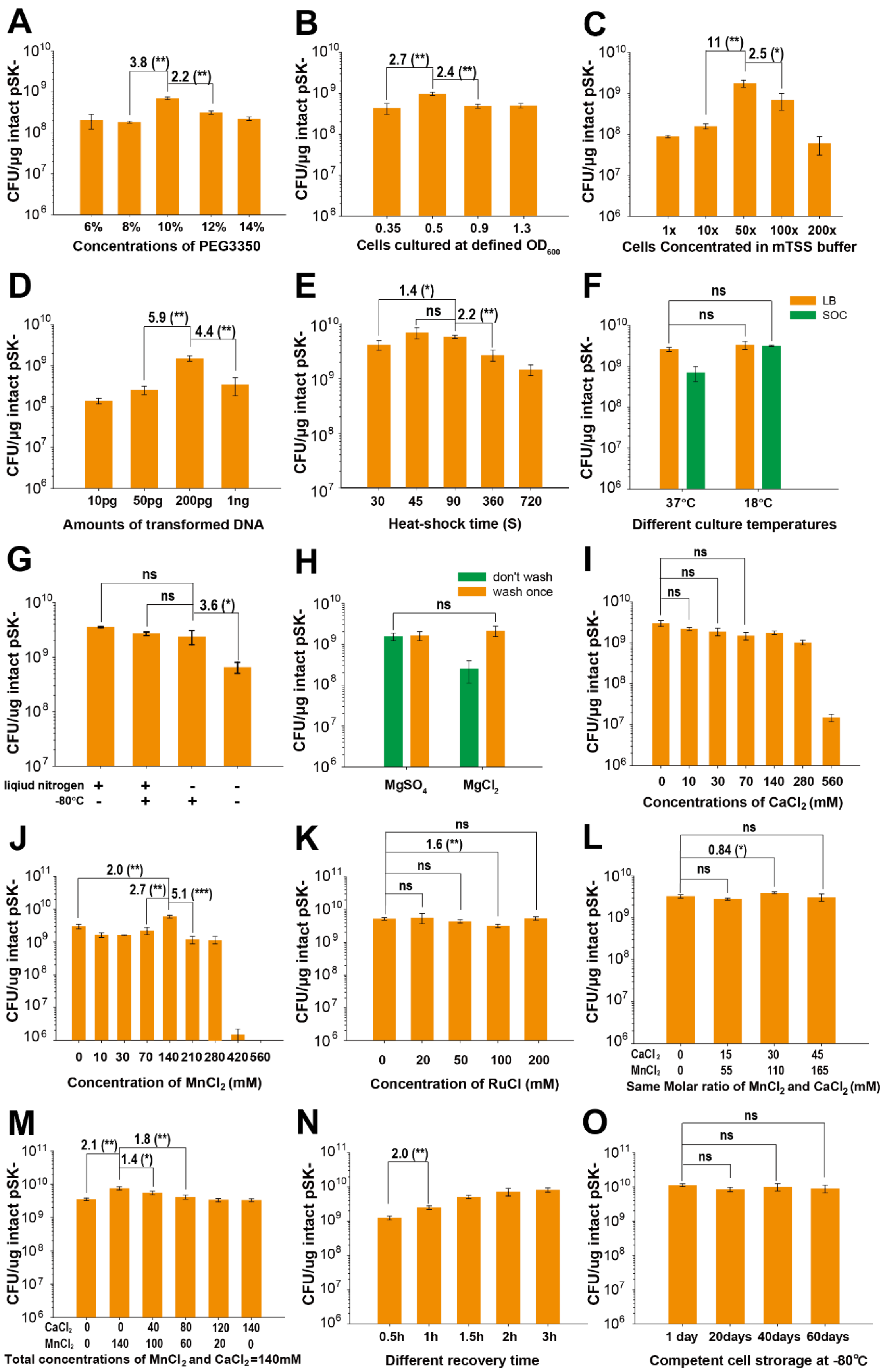

Supplementary Figure S1. The effect of TSS modification (TSS-HI) on TE of

BW3KD. The components and operating procedures in TSS were optimized, including concentrations of PEG3350 (A), cultured stages (OD_{600nm}) of BW3KD (B), concentrated cells in folds (C), amounts of plasmid pSK- (D), heat-shock treatment (E), the temperature and medium used to culture cells (F), -80ºC frozen (G), $MgCl_2$ instead of $MgSO_4$ (H), the addition of Ca^{2+} and Mn^{2+} ions (I-M), and the recovery time (N). The stability of competent cells after storage at -80ºC (O). Data are averages of three samples with standard deviations (error bars) (n=3). The one-way ANOVA method was performed to calculate the p-values (* = $p < 0.05$; ** = $p < 0.01$; *** = $p < 0.001$). The numbers before the brackets represent the difference in folds.

Supplementary Table S1. The strains and plasmids used in this study

Strains and Plasmids	Functions and features	Origin
***E. coli* strains**		
XL1-Blue MRF’	Δ(*mcrA*)183 Δ(*mcrCB-hsdSMR-mrr*)173 *endA1 supE44 thi-1 recA1 gyrA96 relA1* lac *[F’ proAB* $lacI^qZ\Delta M15$ *Tn10* (Tet^r)*]*	Agilent
BW25113	$LacI^+$ $rrnB_{T14}$ $\Delta lacZ_{WJ16}$ *hsdR514* $\Delta araBAD_{AH33}$ $\Delta rhaBAD_{LD78}$ *rph-1 Δ(araB–D)567 Δ(rhaD–B)568 ΔlacZ4787(::rrnB-3) hsdR514 rph-1*	CGSC
Mach1	Str.W Δ*recA1398 endA1 fhuA* Φ80Δ(*lac*)M15 Δ(*lac*)*X*74 hsdR(r_K^- m_K^+)	Invitrogen
Omnimax2	F′[$proAB^+$ $lacI^q$ *lacZΔM15 Tn10*(Tet^R) Δ(*ccdAB*)] *mcrA* Δ(*mrr-hsdRMS-mcrBC) φ80(lacZ)ΔM15 Δ(lacZYA-argF)U169 endA1 recA1 glnV44 thi-1 gyrA96*(Nal^R) *relA1 tonA panD*	Invitrogen
Stbl2	*F- endA1 glnV44 thi-1 recA1 gyrA96 relA1 Δ(lac-proAB) mcrA Δ(mcrBC-hsdRMS-mrr)* λ^-	Invitrogen
Stbl3	F^- *mcrB mrr hsdS20* (r_B^-, m_B^-) *recA13 supE44 ara-14 galK2 lacY1 proA2 rpsL20* (Str^R) *xyl-5* λ^- *leu mtl-1*	Invitrogen
BW2K	Derived from BW25113, ΔendA, ΔfhuA,	This study
BW3KD	Derived from BW25113, ΔendA, ΔfhuA, ΔdeoR	This study
**Plasmids**		
pBluescript SK^-	Abbreved as pSK-, pUC18 ori, Amp^R, used to test TE of competent cells	Stratagene
pCCESAC2	12.4kb, Amp^R, used to test the TE for plasmid with large size	Haoxin Wang’s lab
pSET152-BC	19.4kb, Amp^R, used to test the TE for plasmid with large size	Haoxin Wang’s lab
pSET152-ABC	25.7kb, Amp^R, used to test the TE for plasmid with large size	Haoxin Wang’s lab
pCCESAC2-5H11	43.5kb, Amp^R, used to test the TE for plasmid with large size	Haoxin Wang’s lab
pCCESAC2-M1464	54.6kb, Amp^R, used to test the TE for plasmid with large size	Haoxin Wang’s lab
pCCESAC2-5H11-4H5	75.1kb, Amp^R, used to test the TE for plasmid with large size	Haoxin Wang’s lab
pCC1FOS	8.1kb, Cmr^R, used to test the clone efficiency for large fragments	Epicentre Biotechnologies
pSK::P5tac-phbCAB	derived from pSK-, *phbCAB* genes under the control of 5 tac promoters (P5tac)	This study
pSK::Pkat-eGFP	derived from pSK-, eGFP genes under the control of Pkat promoter	This study

pBAC-λ10K	Plasimd pCC1FOS cloned with 10kb λ phage DNA，used to test the assembly efficiency of large fragments	This study
pBAC-λ15K	Plasimd pCC1FOS cloned with 15kb λ phage DNA，used to test the assembly efficiency of large fragments	This study
pBAC-λ20K	Plasimd pCC1FOS cloned with 20kb λ phage DNA，used to test the assembly efficiency of large fragments	This study

56

Supplementary Table S2. The oligoes used in this study

NO.	Primers	SEQUENCES	Purposes and characteristics
1.	psk-kan-F	GCGGGACTCTGGGGTTCGAAATG	Primers used amplify linearized vector with pSK-Kan (1) as template, which was used for pSK::Pkat-eGFP cloning
2.	psk-kan-R	GCGAAACGATCCTCATCCTGTCTCT	
3.	Pkat-eGFP-F30	GGCGGCCGCTCTAGAACTAGTGGATCCCCCACTGGGCTATCTGGACAAGGG	Primers used amplify Pkat-eGFP fragment with SmaI treated pSK::Pkat-eGFP (1) as template, which was used for pSK::Pkat-eGFP cloning with 30 bp homologous ends
4.	Pkat-eGFP-R30	GATAAGCTTGATATCGAATTCCTGCAGCCCGCATTCTGCCGACATGGAA	
5.	pSK::phbCAB-fr	GGGCTGCAGGAATTCGATATCAAG	Primers used amplify linearized vector with p5TG-phbCAB (1)as template, which was used for pSK::p5TG-phbCAB cloning
6.	pSK::phbCAB-rev	GGGGGATCCACTAGTTCTAGAG	
7.	5PtacCAB-A1-F	CTAGAACTAGTGGATCCCCCTTATGGCGGGTCTGCTATGTGGTGC	Primers used amplify phbCAB-A fragment with p5TG-phbCAB as template, which was used for pSK::p5TG-phbCAB cloning with 20bp homologous ends
8.	5PtacCAB-A2-R	CGAGCACGTTGATCTTGTCCTGGCCGCTGATG	
9.	5PtacCAB-A1-F	CTAGAACTAGTGGATCCCCCTTATGGCGGGTCTGCTATGTGGTGC	Primers used amplify phbCAB-A1 fragment with p5TG-phbCAB as template, which was used for for pSK::p5TG-phbCAB cloning with 20bp homologous ends
10.	5PtacCAB-A1-R	CATGTAGCGCTGCTGGATATCACC	
11.	5PtacCAB-A2-F	GATATCCAGCAGCGCTACATGAAGGACT	Primers used amplify phbCAB-A2 fragment with p5TG-phbCAB as template, which was used for for pSK::p5TG-phbCAB cloning with 20bp homologous ends
12.	5PtacCAB-A2-R	CGAGCACGTTGATCTTGTCCTGGCCGCTGATG	
13.	5PtacCAB-B1-F	GGACAAGATCAACGTGCTCGGCTTCTGCG	Primers used amplify phbCAB-B fragment with p5TG-phbCAB as template, which was used for for pSK::p5TG-phbCAB cloning with 20bp homologous ends
14.	5PtacCAB-B2-R	GTCGACGATCATGGTGTCGACCAGCTTGGC	
15.	5PtacCAB-B1-F	GGACAAGATCAACGTGCTCGGCTTCTGCG	Primers used amplify phbCAB-B1 fragment with p5TG-phbCAB as template, which was used for for pSK::p5TG-phbCAB cloning with 20bp homologous ends
16.	5PtacCAB-B1-R	CGCATCGTTAGTCCAGTGGCT	

17.	5PtacCAB-B2-F	GCCACTGGACTAACGATGCG	Primers used amplify phbCAB-B2 fragment with p5TG-phbCAB as template, which was used for for pSK::p5TG-phbCAB cloning with 20bp homologous ends
18.	5PtacCAB-B2-R	GTCGACGATCATGGTGTCGACCAGCTTGGC	
19.	5PtacCAB-C1-F	TCGACACCATGATCGTCGACGGCCTGTGG	Primers used amplify phbCAB-C fragment with p5TG-phbCAB as template, which was used for for pSK::p5TG-phbCAB cloning with 20bp homologous ends
20.	5PtacCAB-C2-R	ATATCGAATTCCTGCAGCCCCAGCTATGAGCTACTCATATGGCGGCC	
21.	5PtacCAB-C1-F	TCGACACCATGATCGTCGACGGCCTGTGG	Primers used amplify phbCAB-C1 fragment with p5TG-phbCAB as template, which was used for for pSK::p5TG-phbCAB cloning with 20bp homologous ends
22.	5PtacCAB-C1-R	CGCTGAGTCATGTCCACTCCTTGAT	
23.	5PtacCAB-C2-F	GAGTGGACATGACTCAGCGCATTGC	Primers used amplify phbCAB-C2 fragment with p5TG-phbCAB as template, which was used for for pSK::p5TG-phbCAB cloning with 20bp homologous ends
24.	5PtacCAB-C2-R	ATATCGAATTCCTGCAGCCCCAGCTATGAGCTACTCATATGGCGGCC	
25.	5PtacCAB-A1-F	CTAGAACTAGTGGATCCCCCTTATGGCGGGTCTGCTATGTGGTGC	Primers used amplify phbCAB-AB fragment with p5TG-phbCAB as template, which was used for for pSK::p5TG-phbCAB cloning with 20bp homologous ends
26.	5PtacCAB-B2-R	GTCGACGATCATGGTGTCGACCAGCTTGGC	
27.	5PtacCAB-A1-F	CTAGAACTAGTGGATCCCCCTTATGGCGGGTCTGCTATGTGGTGC	Primers used amplify phbCAB-ABC fragment with p5TG-phbCAB as template, which was used for for pSK::p5TG-phbCAB cloning with 20bp homologous ends
28.	5PtacCAB-C2-R	ATATCGAATTCCTGCAGCCCCAGCTATGAGCTACTCATATGGCGGCC	
29.	5PtacCAB-B1-F	GGACAAGATCAACGTGCTCGGCTTCTGCG	Primers used amplify phbCAB-BC fragment with p5TG-phbCAB as template, which was used for for pSK::p5TG-phbCAB cloning with 20bp homologous ends
30.	5PtacCAB-C2-R	ATATCGAATTCCTGCAGCCCCAGCTATGAGCTACTCATATGGCGGCC	
31.	pBAC-fr-λ	AAGCTTGAGTATTCTATAGTCTCACC	Primers used amplify linearized vector with pCC1FOS as template,

32.	pBAC-rev-λ	GTATTACAATTCACTGG<br>CCGTCGT	which was used for pBAC-λ10K,<br>pBAC-λ15K, pBAC-λ20K cloning
33.	λ10K-fr-pBAC	CGGCCAGTGAATTGTAA<br>TACTCGGTGCGAGTATC<br>CGTACCATTCAGA	Primers used amplify 10kb fragment<br>with λ phage genome as template,<br>which was used for pBAC-λ10K<br>cloning with 20bp homologous ends
34.	λ10K-rev-pBAC	GGTGAGACTATAGAAT<br>ACTCAAGCTTTCGTCTG<br>CCGTGCTGATCTCCT	
35.	λ15K-fr-pBAC	CGGCCAGTGAATTGTAA<br>TACTCGGTGCGAGTATC<br>CGTACCATTCAGA	Primers used amplify 15kb fragment<br>with λ phage genome as template,<br>which was used for pBAC-λ15K<br>cloning with 20bp homologous ends
36.	λ15K-rev-pBAC	ACTATAGAATACTCAAG<br>CTTCTGGGCAACCATGT<br>TATCCAGTGAG	
37.	λ20K-fr-pBAC	CGGCCAGTGAATTGTAA<br>TACTCGGTGCGAGTATC<br>CGTACCATTCAGA	Primers used amplify 20kb fragment<br>with λ phage genome as template,<br>which was used for pBAC-λ20K<br>cloning with 20bp homologous ends
38.	λ20K-rev-pBAC	ACTATAGAATACTCAAG<br>CTTGCACTTGATGATGC<br>GTTCGTTTCTGA	

Reference

1. Xia YZ, Li K, Li JJ, Wang TQ, Gu LC, Xun LY. 2019. T5 exonuclease-dependent assembly offers a low-cost method for efficient cloning and site-directed mutagenesis. Nucleic Acids Research 47:e15.
